# Phylogenetic Status and Timescale for the Diversification of *Steno* and *Sotalia* Dolphins

**DOI:** 10.1371/journal.pone.0028297

**Published:** 2011-12-07

**Authors:** Haydée A. Cunha, Lucas C. Moraes, Bruna V. Medeiros, José Lailson-Brito, Vera M. F. da Silva, Antonio M. Solé-Cava, Carlos G. Schrago

**Affiliations:** 1 Departamento de Genética, Instituto de Biologia, Universidade Federal do Rio de Janeiro, Rio de Janeiro, Brazil; 2 Laboratório de Mamíferos Aquáticos e Bioindicadores, Faculdade de Oceanografia, Universidade do Estado do Rio de Janeiro, Rio de Janeiro, Brazil; 3 Laboratório de Mamíferos Aquáticos, Instituto Nacional de Pesquisas da Amazônia, Amazonas, Brazil; J. Craig Venter Institute, United States of America

## Abstract

Molecular data have provided many insights into cetacean evolution but some unsettled issues still remain. We estimated the topology and timing of cetacean evolutionary relationships using Bayesian and maximum likelihood analyses of complete mitochondrial genomes. In order to clarify the phylogenetic placement of *Sotalia* and *Steno* within the Delphinidae, we sequenced three new delphinid mitogenomes. Our analyses support three delphinid clades: one joining *Steno* and *Sotalia* (supporting the revised subfamily Stenoninae); another placing *Sousa* within the Delphininae; and a third, the Globicephalinae, which includes *Globicephala*, *Feresa*, *Pseudorca*, *Peponocephala* and *Grampus*. We also conclude that *Orcinus* does not belong in the Globicephalinae, but *Orcaella* may be part of that subfamily. Divergence dates were estimated using the relaxed molecular clock calibrated with fossil data. We hypothesise that the timing of separation of the marine and Amazonian *Sotalia* species (2.3 Ma) coincided with the establishment of the modern Amazon River basin.

## Introduction

The phylogeny of cetaceans has been intensively investigated over the last decade using molecular data. Classical arrangements have been drastically modified, such as the positioning of the clade within the artiodactyls [1], [2], [3], [4], [5], [6], [7], [8] and the monophyletic status of several genera and higher taxonomic groups [9], [10], [11], [12], [13], [14]. Modern Cetacea consists of two evolutionary lineages supported by morphological [15], [16] and molecular data [11], [17]: the Mysticeti (baleen whales) and the Odontoceti (toothed whales). Odontocetes are of particular evolutionary interest as they include several species that have adapted to riverine environments. Furthermore, the rapid diversification of the Delphinidae makes the phylogenetic inference of their evolutionary history challenging.

Within the Delphinidae, the systematics of the genus *Sotalia* has been the focus of several recent studies. After the recognition that the genus comprises two species, *S. fluviatilis* and *S. guianensis*, the former became the only known exclusively freshwater delphinid in the world [18]. However, the phylogenetic placement of *Sotalia* within the family is still unresolved [5], [10], [19], [20], [21], [22]. Moreover, different studies have estimated different timings for the separation between the two *Sotalia* species [20], [21], [23], [24]. Although it is generally believed that the changes in the Amazon during the Plio-Pleistocene drove the diversification of *Sotalia* species [18], [23], a clearer evolutionary scenario can only be depicted in light of reliable estimates of the phylogenetic position and the chronology of *Sotalia* speciation.

In order to better assess such historical information on *Sotalia* evolution and to establish its phylogenetic position within the Delphinidae, we sequenced the complete mitochondrial genomes of *S. fluviatilis*, *S. guianensis* and *Steno bredanensis*. Besides providing a more precise estimate of the timing of separation of *Sotalia* species, our analyses also shed light on delphinid phylogeny and increased the evidence in favour of *Steno* being a sister taxon to *Sotalia*.

## Results

**Figure 1:**
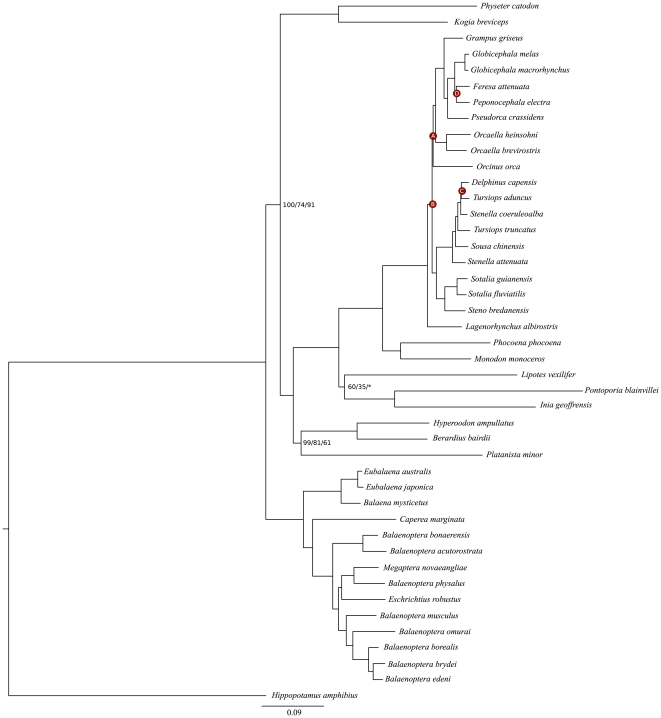
Phylogeny of Cetacea. Support values represent PP/aLRT/BS. Nodes without information were supported by 100/100/100. (A) 61/52/*, (B) 100/80/*, (C) 55/89/*, (D) 100/91/73. (*) Indicates that RAxML BS is <50.

Both Bayesian and maximum likelihood trees were topologically congruent and presented a similar pattern of statistical support distribution for the nodes (Figure 1). Except for delphinid relationships, our phylogeny is largely in agreement with recent mitogenomic studies [11], [25], [26], [27], [28].

Within the Delphinidae, two major lineages were statistically supported (100% PP, aLRT and BS); the Delphininae (*Tursiops*+*Stenella*+*Delphinus*+*Sousa*) and the Stenoninae (*Sotalia*+*Steno*) clades. Those two clades are closely related (100% PP and aLRT, 99% BS). A third clade, the Globicephalinae (*Globicephala*+*Pseudorca*+*Grampus+Peponocephala*+*Feresa*), may include *Orcaella*, and is only supported with the exclusion of *Orca*. Alternatively, subfamily Orcaellinae could be a sister taxon to Globicephalinae. There is no support for subfamily Orcininae (*Orcinus*+*Orcaella*). The position of *Orcinus* within the family is unclear, and the white-beaked dolphin, *Lagenorhynchus albirostris*, was inferred as a sister to the remaining delphinids (100% PP, aLRT and BS).

Contrary to recent works of Caballero *et al.* [19] and McGowen *et al.* [21], [22], our analysis of complete dolphin mitochondrial genomes show that *Steno bredanensis* is phylogenetically more related to *Sotalia* dolphins than to the Globicephalinae. We have investigated whether the arrangement proposed by the former papers [19], [21], [22] was statistically superior to the one supported by our tree via the Kishino-Hasegawa test [29]. The topology presented in this study (Figure 1) significantly increases the likelihood of our data (ΔlnL = 339.0, p≅0), rejecting the null hypothesis that the likelihoods of both trees are equal.

**Figure 2:**
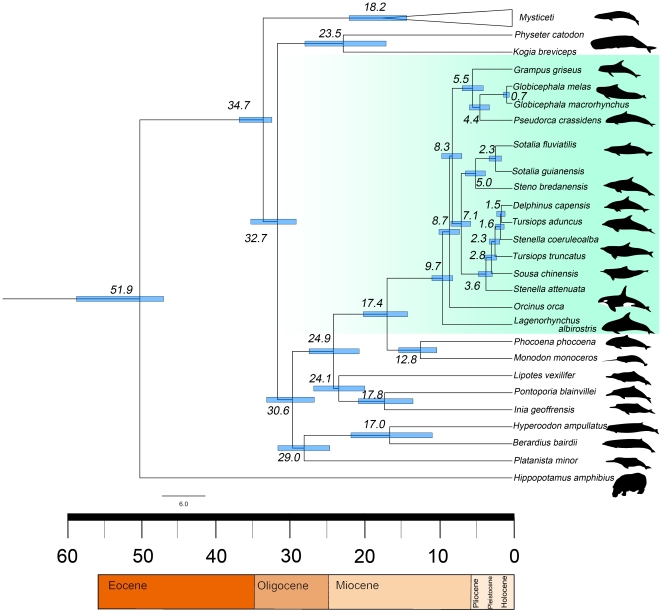
Timescale of Odontoceti evolution.

Beginning in the late Miocene (9.5±1.4 Ma, *Mega annum*) the Delphinidae experienced a rapid diversification (Figure 2). The clades that presented significant statistical support within the Delphinidae diversified around the Pliocene (5.5 – 3.5 Ma). The separation between the marine and riverine species of *Sotalia* was estimated at approximately 2.3 Ma (1.3–3.4 Ma). Species with difficult taxonomic assignment at the generic level, such as *Tursiops* spp. and *Stenella* spp., were all inferred to have diversified in the late Pliocene and Pleistocene.

## Discussion

Mitogenomic analyses have the potential to disclose delphinid evolutionary relationships because the group has undergone rapid and recent diversification. The effective population size of mitochondrial lineages is smaller than that of nuclear gene lineages. Thus, the probability of reaching reciprocal monophyly in small time intervals is higher in mitochondrial genomes [30]. As more cetacean mitogenomes are sequenced, phylogenetic reconstructions become more capable of settling long standing issues. The inclusion of three new dolphin mitogenomes in the analyses have contributed to the resolution of uncertain delphinid evolutionary affinities, especially concerning the phylogenetic placement of *Sotalia* and *Steno* dolphins.

### Delphinidae

The grouping of *Sotalia* and *Steno* was strongly supported, as well as the positioning of *Sousa* within the Delphininae. These results indicate that *Steno* is the sister group of *Sotalia*, thus supporting the revised subfamily Stenoninae as proposed by LeDuc *et al.* [10]. Although those authors could not reach a definitive conclusion regarding the sister group relationship between *Steno* and *Sotalia* based on their cytochrome b data, they decided to maintain both genera in Stenoninae as indicated by earlier morphological studies. However, they suggested that the revised Stenoninae does not include *Sousa*, a genus also traditionally assigned to that subfamily based on morphology. Instead, they placed *Sousa* in Delphininae. Our phylogeny reinforces the view that *Sousa* belongs in the Delphininae, as formerly indicated by other studies [5], [19], [20], [21], [25], [28].

The close affinity between *Steno* and *Sotalia* and the placement of *Sousa* in the Delphininae were previously suggested using cytochrome b data [5], [31] and a supermatrix of nine nuclear and six mitochondrial genes [20]. Three studies disputed this conclusion. Caballero *et al.* [19], and McGowen *et al.* [21], [22] proposed placing both *Sotalia* and *Sousa* in the Delphininae, and grouping *Steno* with Globicephalinae+*Grampus*+*Orcaella*. In those studies, conclusions were based on a combined nuclear and mitochondrial dataset. In the case of Caballero *et al.* [19] and McGowen *et al.* [21], [22], the combined phylogeny seemed to be driven mainly by their nuclear data, since their mitochondrial phylogeny did not support such groupings. Interestingly, however, Steeman *et al.* [20] also combined nuclear and mitochondrial data, and recovered the same topology observed here and in the studies mentioned above. In a recent mitogenomic phylogeny of the Delphinidae *Steno* was also placed outside the Globicephalinae [28].

The new conformation of Stenoninae implies that the phylogenetic placement of the fossil genus *Astadelphis* requires revision. *Astadelphis* has been assigned to this subfamily, and is only recorded from Pliocene deposits (3.1–3.8 Ma) of Italy. It has been considered by different authors as phylogenetically close to *Steno* and *Sotalia* [32] or to *Sousa* [33]. If that latter view is correct, *Astadelphis* does not belong in Stenoninae.

Our phylogeny confirms the phylogenetic position of *Grampus* within the Globicephalinae, as first proposed by LeDuc *et al.* [10] and contrary to the traditional view, based on morphology, which included *Grampus* in the subfamily Delphininae [34]. Previous studies based on the cytochrome b [5], mitochondrial genomes [28], [35] and combined mitochondrial and nuclear genes [19], [20], [21], [22], [36] also recovered the placement of *Grampus* in the Globicephalinae.

On the other hand, *Orcinus*, traditionally placed in the Globicephalinae [34], usually figures in molecular phylogenies either as “incertae sedis” [10], [20], [21], [36], or pooled with *Orcaella* [5]. Recently, however, a mitogenomic study proposed the inclusion of both *Orcinus* and *Orcaella* in the Globicephalinae [28], in agreement with early views based on morphology. Our phylogeny strongly supports the exclusion of *Orcinus* from Globicephalinae. We also refute the existence of the subfamily Orcininae (*Orcinus*+*Orcaella*), as proposed by LeDuc *et al.* [10] and Agnarsson and May-Collado [5] based on cytochrome b data. Instead, *Orcaella* may be part of the Globicephalinae, or warrant a separate subfamily, as proposed by Perrin [34]. The relationship of *Orcinus* to the other delphinid subfamilies remains unresolved.

*Lagenorhynchus albirostris* occupies the most basal position within the delphinids. Unfortunately, since our phylogeny lacks mitogenomes from the genera *Lissodelphis*, *Cephalorhynchus* and the other *Lagenorhynchus* species, it is not yet possible to ascertain the evolutionary relationships of all Delphinidae subfamilies, nor the monophyly of Lissodelphininae, which has been questioned [10], [37].

Unsurprisingly, our analyses were unable to shed light on the *Stenella-Delphinus-Tursiops* complex, due to the current lack of mitochondrial genomes from many species. Our phylogeny only reinforced previous findings concerning the para- or polyphyly of *Stenella* and *Tursiops* [5], [10], [20], [21]. The recent speciation (<4 Ma) of these lineages poses many difficulties in phylogenetic inference (such as low number of informative characters, incomplete lineage sorting and the possible existence of fertile hybrids), and understanding their evolution may require the aid of phylogeographical approaches.

### Timing of *Sotalia* speciation

Dating the divergence between riverine and marine *Sotalia* is crucial to understand the phylogeography of *S. fluviatilis*, as it indicates when this species became genetically isolated after colonising the Amazon basin. To date, all divergence estimates have been based either on the mitochondrial control region [23], cytochrome b [20], [21] or on both markers [18], [24]. Thus, our mitogenomic phylogeny provides the best opportunity so far to date more precisely the divergence between *S. guianensis* and *S. fluviatilis*.

Four previous studies attempted to estimate the timing of *Sotalia* speciation. The genetic divergence (*p* distance) between the *Sotalia* species observed by Cunha *et al.* [18] for both the control region and the cytochrome b was 2.5%. Taking into consideration the evolutionary rates of these markers in cetaceans – control region: 0.5% to 1% per million years [38]; cytochrome b: 1%/Ma, [39] – the speciation event that separated both lineages would have happened between 2.5 and 1.25 Ma, during the early Pleistocene [24]. A somewhat similar estimate was obtained using a relaxed molecular clock and cytochrome b data, 1.99 Ma (0.63–3.67) [21]. The dates proposed by Cunha *et al.* [24] and McGowen *et al.* [21] overlap with our estimate – 2.3 Ma (1.3–3.4). A different timing of the speciation was proposed by Caballero *et al.* [23], who calibrated a molecular clock for the control region using the estimated divergence between *Sotalia* and *Phocoena phocoena* based on the fossil record (10 to 11 Ma). Therefore, they arrived at a faster substitution rate, and dated the divergence between *S. fluviatilis* and *S. guianensis* much later, at 1.0 to 1.2 Ma. Finally, the oldest time estimate for the separation of *Sotalia* species (3.5 Ma) was obtained by Steeman *et al.* [20]. It is noteworthy that, in spite of being a supermatrix analysis (of 15 mitochondrial and nuclear markers) using seven fossil calibration points and relaxed clock models, the divergence between the *Sotalia* species in that study was based exclusively on the cytochrome b gene (the only marker analysed from *S. guianensis* by the authors).

Besides being the most precise estimate available, our dating coincides remarkably well with the establishment of the modern Amazon River basin. Until recently, authors accepted that the modern Amazon basin was already established in the Miocene, but that view has changed based on new geological data. Sediment analyses showed that the Amazon River only attained its present conformation by the beginning of the Pleistocene, approximately 2.5 Ma [40], [41]. At the same time, there was a major lowering of sea level from 3 to 2 Ma [42], which could have been partly responsible for changing the river's course eastwards, coupled with Andean tectonics [40], [41]. Irrespective of the environmental conditions prevailing at the time, *Sotalia* dolphins that colonised the Amazon basin certainly had an Atlantic origin, because the connection with the Caribbean via the Paleo-Orinoco river and the Paleo-Maracaibo had been closed since the rising of the northern Andes cordillera, 8 Ma [43], [44].

## Methods

### DNA extraction, amplification and sequencing

Total DNA was isolated from skin samples of *Steno bredanensis*, *Sotalia guianensis* and *S. fluviatilis* using the standard phenol-chloroform procedure [45]. Two long fragments (about 9 Mb and 7 Mb), comprising the entire mitochondrial genome, were PCR-amplified using the primers described by Sasaki *et al.* [46]. Amplifications were carried out in 50 µL reactions using the Qiagen LongRange PCR Kit. Reagent concentrations and cycling profile followed the manufacturer's instructions.

Long-PCR products were then used as templates for amplification of smaller fragments, using the primers described in Xiong *et al.* [25] and others developed in this study (Table S1). PCR reactions (30 µL) contained 1.5 U Taq, 200 µM dNTP, 2.5 mM MgCl_2_, 15 µg BSA and 0.5 µM of each primer. Amplification thermal conditions were as follows: 3 min at 93°C, 30 cycles of 1 min at 92°C, 1 min at 50°C or 55°C, 1 min at 72°C, and 5 min of final extension at 72°C. PCR products were purified using ExoSap (GE) and both strands were sequenced in an ABI3130 using BigDye chemistry.

Sequences were edited in SeqMan 7 (DNAStar), using the complete mitochondrial genome of *Sousa chinensis* [GenBank EU557091] as template to build the contigs. The complete mitogenomes were deposited in GenBank (JF681038, JF681039 and JF681040).

### Alignment and evolutionary analyses

All cetacean mitochondrial genomes available in GenBank as of March 2011 were included in this study (Table S2). Alignments were conducted for each gene individually in ClustalW [47] and manually checked. A supermatrix of 15,873 bp was used in phylogeny estimation and divergence time inference. It consisted of the concatenation of all 13 protein coding genes, tRNA and rRNA genes and D-loop. Protein coding genes were further separated into three partitions containing only first, second and third codon positions. This was done to maximise rate variation among partitions [48]. Phylogeny estimation was performed in MrBayes 3 [49], PhyML 3 [50] and RAxML 7.0.4 [51]. In MrBayes and RAxML, each partition was allowed to evolve independently under the GTR+G+I model.

For the Bayesian inference, the Markov chain Monte Carlo (MCMC) settings used were as follows. Two independent runs, with three Markov chains each, were sampled every 100^th^ generation during 10^7^ generations, resulting in 100,000 trees in each run, of which 25% were discarded as burn-in. In RAxML, maximum likelihood (ML) topology estimation was conducted independently from 200 different starting trees. Then, 1,000 bootstrap pseudoreplicates were run to obtain the statistical support for the nodes of the tree with the highest log-likelihood. ML tree search in PhyML was performed by the SPR algorithm and the aLRT statistic [52] was used to evaluate node confidence.

Divergence time inference was conducted in BEAST 1.6.1 [53] using the same data partitioning, substitution model and MCMC settings described above. Substitution rate evolution was modelled by the uncorrelated lognormal distribution. Tree topology prior followed the Yule process.

### Calibration information used as priors

**Figure 3:**
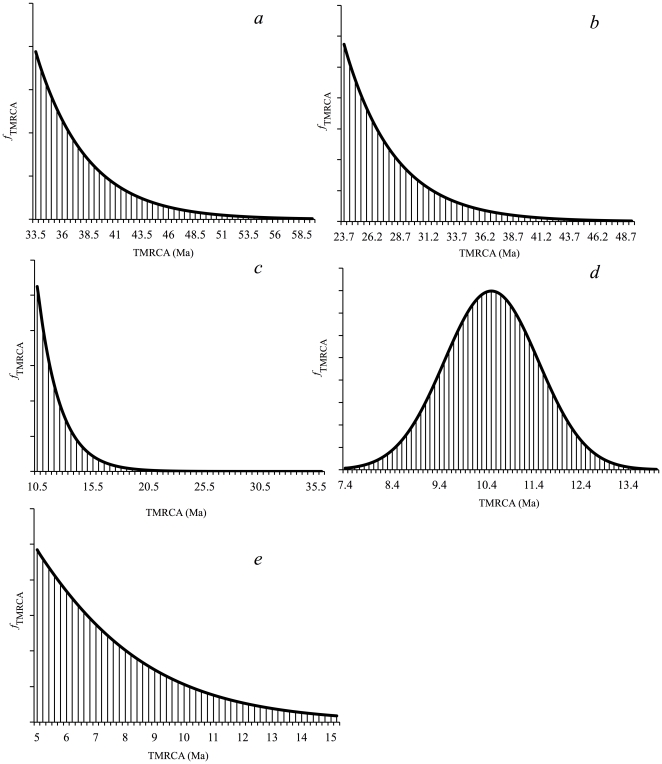
Calibration information used as priors in Bayesian dating analyses. (a) TMRCA of modern Cetacea; (b) TMRCA of Odontoceti; (c) Age of the *Monodon*/*Phocoena* split; (d) Age of the Delphinidae diversification; (e) Age of the Iniidae/Pontoporidae divergence.

The ages of five nodes were constrained by calibration information based on the fossil record of cetaceans (Figure 3):

1. The time since the most recent common ancestor (TMRCA) of Cetacea was calibrated by a gamma prior with shape = 1.0, scale = 4.8 and offset = 33.5 Ma. This is based on the early Mysticeti fossils at the Eocene/Oligocene boundary [54], [55]. The gamma prior was adjusted so that the tail of the distribution would include the Archaeoceti fossils, which are supposedly the stem cetacean lineage.
2. The earliest members of the Odontoceti [56] were found in the late Oligocene [57]. By around 23.7 Ma, odontocetes had already diversified, because this is the age of *Ferecetotherium*, which presents autapomorphies of the Physeteridae [16]. We have used a gamma prior with shape = 1, scale = 4.5 and offset = 23 Ma. The tail of the gamma distribution was extended to the Eocene, to safely incorporate stem lineages of the mysticetes and odontocetes.
3. The age of the *Monodon*/*Phocoena* split was constrained by a gamma prior with shape = 1, scale = 2 and offset = 10.5 Ma. Prior information was based on the oldest fossil Phocoenidae, *Salumiphocaena stocktoni*, from the late Miocene of North America [58]. The shape of the distribution was set in order to accommodate the Miocene epoch.
4. The diversification of Delphinidae was constrained by a normal prior with mean = 10.5 and standard deviation = 1.0 Ma, which resulted in a 95% interval from 8.5 to 12.5. This prior was based on the record of early delphinids from the late Miocene [59].
5. The divergence between Iniidae and Pontoporiidae had already taken place in the early Pliocene. We used a gamma prior with shape = 1.7, scale = 2.5 and offset = 5 Ma to estimate the TMRCA of *Inia*/*Pontoporia*. Xiong *et al.* [25] used the fossil *Brachydelphis* to constrain the *Inia*/*Pontoporia* divergence. However, the extensive morphological revision by Geisler and Sanders [16] places *Brachydelphis* as stem lineage of “Platanistoidea”, which includes extant *Lipotes* and *Platanista* as well as *Inia* and *Pontoporia*. Thus, we chose not to consider this fossil as a stem Pontoporiidae and established an offset for the gamma prior at 5 Ma, which safely includes late Miocene South American fossils of iniids and pontoporiids [60].

## Supporting Information

Table S1 **Additional primers designed to enable complete sequencing of the delphinid mitochondrial genome.**
(DOC)

Table S2 **Accession numbers of the species used in this study.**
(DOC)
