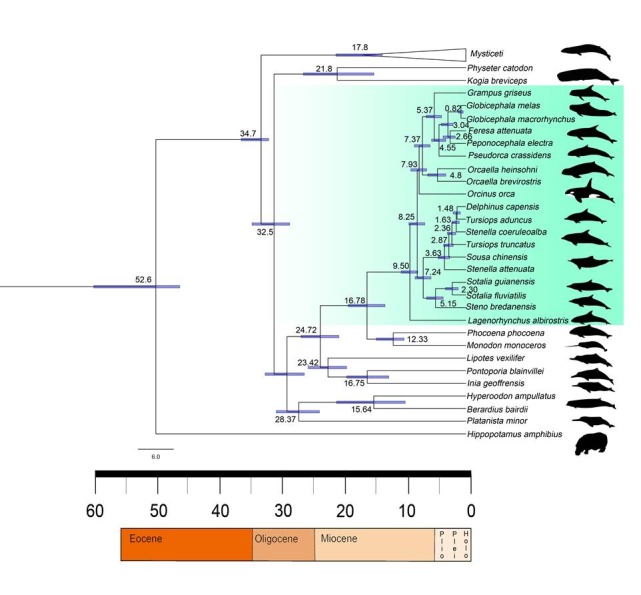# Correction: Phylogenetic Status and Timescale for the Diversification of *Steno* and *Sotalia* Dolphins

**DOI:** 10.1371/annotation/e624380d-1b9c-4134-a68d-83629fbf26e1

**Published:** 2012-08-09

**Authors:** Haydée A. Cunha, Lucas C. Moraes, Bruna V. Medeiros, José Lailson-Brito, Vera M. F. da Silva, Antonio M. Solé-Cava, Carlos G. Schrago

There was an error in Figure 2. The correct Figure 2 can be viewed here: 

**Figure:**