# Supplementary material for: Phylogenetic Status and Timescale for the Diversification of Steno and Sotalia Dolphins
Source: PLoS One. 2011 Dec 7;6(12):e28297. doi: 10.1371/journal.pone.0028297 (PMC3233566; doi:10.1371/journal.pone.0028297)
Supplement: Table S1 — Additional primers designed to enable complete sequencing of the delphinid mitochondrial genome. (DOC) [file pone.0028297.s001.doc]

Table S1: Additional primers designed to enable complete sequencing of the delphinid mitochondrial genome.

| ***Primer name***  ***(Target fragment)*** | ***Forward primer (5’ – 3’)*** | ***Reverse primer (5’ – 3’)*** |
| --- | --- | --- |
| mt12 (partial 12S RNA) | TTACACATGCAAGCATCCGC | GGTACTCTCTCTATAGCGCC |
| mt13 (ND1) | TCAGAACTCGTATCTGGC | ATTAGTCCTGTGCTTAGGG |
| mt14 (ND2) | ATACTCCTACCCCTCACACC | TATAAAGCTGGTCGTCTCCG |
| mt15 (COX2 – ATP6) | CTGAAGCTATGAATATACCG | TATTCCAAGAAGGTTTGTGG |
| mt16 (ND3 – ND4) | CATCAGAATACTATGAAGCC | AAGTGGTTCTTTTAGAAGG |
| mt17 (partial 12S RNA) | GGTTACAGGACATAGTAC | GGTATATAGACTGAAGTAG |
| mt18 (ND5) | ACTCAAGCACAATAGTCGTA | GATGTATGATTGTAGGGAAG |
| mt19 (ND4 – ND5) | GGATGGTGATTCAGTGTCAG | CTACTTGATGACTATTAGCATG |
| mt20 (ND6) | GTAACAATTCTAGGCTTTATTC | GTTGACGTCTCGACAGATG |
