# Supplementary material for: Phylogenetic Status and Timescale for the Diversification of Steno and Sotalia Dolphins
Source: PLoS One. 2011 Dec 7;6(12):e28297. doi: 10.1371/journal.pone.0028297 (PMC3233566; doi:10.1371/journal.pone.0028297)
Supplement: Table S2 — Accession numbers of the species used in this study. (DOC) [file pone.0028297.s002.doc]

Table S2: Accession numbers of the species used in this study.

| **Accession** | **Species** |
| --- | --- |
| NC_005268 | *Balaena mysticetus* |
| NC_005271 | *Balaenoptera acutorostrata* |
| NC_006926 | *Balaenoptera bonaerensis* |
| NC_006929 | *Balaenoptera borealis* |
| NC_006928 | *Balaenoptera brydei* |
| NC_007938 | *Balaenoptera edeni* |
| NC_001601 | *Balaenoptera musculus* |
| NC_007937 | *Balaenoptera omurai* |
| NC_001321 | *Balaenoptera physalus* |
| NC_005274 | *Berardius bairdii* |
| NC_005269 | *Caperea marginata* |
| NC_012061 | *Delphinus capensis* |
| NC_005270 | *Eschrichtius robustus* |
| NC_006930 | *Eubalaena australis* |
| NC_006931 | *Eubalaena japonica* |
| HM060333 | *Globicephala macrorhynchus* |
| HM060334 | *Globicephala melas* |
| NC_012062 | *Grampus griseus* |
| NC_000889 | *Hippopotamus amphibius* |
| NC_005273 | *Hyperoodon ampullatus* |
| NC_005276 | *Inia geoffrensis* |
| NC_005272 | *Kogia breviceps* |
| NC_005278 | *Lagenorhynchus albirostris* |
| NC_007629 | *Lipotes vexillifer* |
| NC_006927 | *Megaptera novaeangliae* |
| NC_005279 | *Monodon monoceros* |
| GU187211 | *Orcinus orca* |
| NC_008830 | *Phacochoerus africanus* |
| NC_005280 | *Phocoena phocoena* |
| NC_002503 | *Physeter catodon* |
| NC_005275 | *Platanista minor* |
| NC_005277 | *Pontoporia blainvillei* |
| JF681040 | *Sotalia fluviatilis* |
| JF681039 | *Sotalia guianensis* |
| NC_012057 | *Sousa chinensis* |
| NC_012051 | *Stenella attenuata* |
| NC_012053 | *Stenella coeruleoalba* |
| JF681038 | *Steno bredanensis* |
| NC_012058 | *Tursiops aduncus* |
| NC_012059 | *Tursiops truncatus* |
